# The microbial abundance dynamics of the paediatric oral cavity before and after sleep

**DOI:** 10.1080/20002297.2020.1741254

**Published:** 2020-03-30

**Authors:** Jessica A. P. Carlson-Jones, Anna Kontos, Declan Kennedy, James Martin, Kurt Lushington, Jody McKerral, James S. Paterson, Renee J. Smith, Lisa M. Dann, Peter Speck, James G. Mitchell

**Affiliations:** a Department of Respiratory and Sleep Medicine, Women’s and Children’s Hospital, Adelaide, Australia; b Robinson Research Institute, School of Paediatrics and Reproductive Health, the University of Adelaide, Adelaide, Australia; c College of Science and Engineering, Flinders University, Adelaide, South Australia, Australia; d School of Psychology, Social Work and Social Policy, University of South Australia, Adelaide, Australia; e College of Medicine and Public Health, Flinders University, Adelaide, South Australia, Australia

**Keywords:** Oral microbiome, sleep, flow cytometry, bacteria, viruses

## Abstract

**Objective**: Microhabitats in the oral cavity differ in microbial taxonomy. However, abundance variations of bacterial and viral communities within these microhabitats are not fully understood.

**Aims and Hypothesis**: To assess the spatial distribution and dynamics of the microbial abundances within 6 microhabitats of the oral cavity before and after sleep. We hypothesise that the abundance distributions of these microbial communities will differ among oral sites.

**Methods**: Using flow cytometry, bacterial and virus-like particle (VLP) abundances were enumerated for 6 oral microhabitats before and after sleep in 10 healthy paediatric sleepers.

**Results**: Bacterial counts ranged from 7.2 ± 2.8 × 10^5^ at the palate before sleep to 1.3 ± 0.2 × 10^8^ at the back of the tongue after sleep, a difference of 187 times. VLPs ranged from 1.9 ± 1.0 × 10^6^ at the palate before sleep to 9.2 ± 5.0 × 10^7^ at the back of the tongue after sleep, a difference of 48 times.

**Conclusion**: The oral cavity is a dynamic numerically heterogeneous environment where microbial communities can increase by a count of 100 million during sleep. Quantification of the paediatric oral microbiome complements taxonomic diversity information to show how biomass varies and shifts in space and time.

## Introduction

Within the oral cavity are numerous microhabitats that provide distinct environments for the colonisation of specific microbial communities [1,2]. By defining the oral microbiome by its microhabitats, a more focused analysis in the spatial distribution of microbial communities in health and disease can be made [2–4]. Previous studies have proposed that the oral microbiome is taxonomically stable over longer periods of time [5–9]. However, Takayasu et al. recently demonstrated that the human oral microbiome followed a circadian cycle, a period of 24 hours [10]. During this time, it was reported that the relative abundance of certain bacterial genera increased during sleep, while others decreased. Whether the overall absolute microbial abundances at each microhabitat within the oral cavity increased or decreased during sleep needs further investigation.

During sleep the oral environment changes due to shifts in saliva flow, pH, temperature, and oxygen availability [4,11–13]. As these factors are involved in shaping the microbial community structure within the oral cavity [14,15], it is likely that the microbial abundance dynamics during sleep will change. With increasing evidence to suggest that microbial dysbiosis within the oral microbiome is linked with chronic disease, it is important to establish the microbial abundance dynamics within healthy states first before pathological states, where the normal sleeping pattern is perturbed, are investigated.

Flow cytometry is an inexpensive method used to enumerate and monitor absolute microbial abundance dynamics within numerous environments [16–23]. This technique has been optimised to count microorganisms, including viruses, within samples that would otherwise be deemed too low for epifluorescence and transmission electron microscopy [16,17,23]. As the majority of bacteria are non-culturable, flow cytometry provides a culture-free enumeration alternative that eliminates enrichment biases [16]. Absolute abundance data adds a new dimension to microbial community analysis compared to relative abundances. It allows for comparison across studies to determine the presence or absence of a potential pathogen, along with the ability to assess whether critical concentrations are required for pathogenesis and what those concentrations are [24–26]. However, the first step is to be able to measure absolute abundance, which frequently applied sequencing techniques are unable to do [24,27].

Given the environmental and taxonomic heterogeneity within the oral cavity [1,2], we hypothesise that the absolute abundance distributions of these microbial communities will also differ among oral sites. In addition, that during sleep, the absolute microbial abundances will increase. Therefore, the aim of this study was to assess the spatial distribution and dynamics of the absolute microbial abundances within 6 microhabitats of the paediatric oral cavity before and after sleep.

## Materials and methods

### Ethics statement

This study was approved by the Human Research Ethics Committees of the Women’s and Children’s Hospital and the University of Adelaide, South Australia. The study was conducted in accordance with the 1964 Declaration of Helsinki and its later amendments. Participants were recruited from the general population of Adelaide children and adolescents through advertising. Parents of participants provided written consent and children written assent for involvement in the study. Parents also completed a child’s health and behaviour questionnaire prior to sample collection. Participants were excluded from the study if they were on medication that affected sleep or respiration, had significant asthma, craniofacial abnormalities or had developmental/psychiatric disorders.

Samples were collected from 10 children and adolescents (male n = 7, female n = 3). All participants underwent an overnight polysomnography sleep test at the Women’s and Children’s Hospital, Adelaide, Australia. Sleep tests were scored using the 2015 American Academy of Sleep Medicine scoring criteria for children [28]. All children were classified as healthy sleepers as determined by an Obstructive Apnoea Hypopnea Index value of less than one. Participants involved in the study were asked to refrain from oral hygiene practices, such as brushing teeth or antimicrobial rinses, for the duration of the study. Ages ranged from 6.08 to 16.58 years (mean 10.3 ± 1.2 years). The average BMI for all participants was 20.1 ± 1.8. Healthy children were chosen for this study to limit the exposure of unhealthy lifestyle factors. This includes smoking and excessive alcohol consumption which are known to influence the oral microbiome [29,30].

### Sample collection

**Figure 1.**
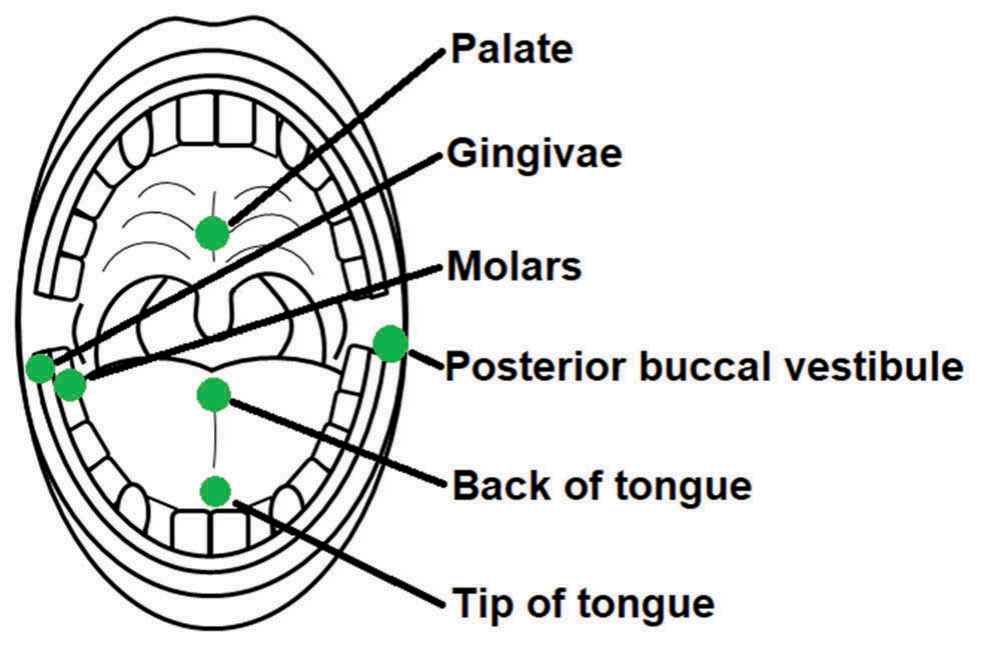
**Microhabitat sample locations in the paediatric oral cavity**. Separate swabs were rotated clockwise 6 times at each location to collect oral samples

Sterile rayon swabs (Copan, Brescia, Italy; product code: 155 C) were used to collect samples from participants. These swabs were individually packaged in their own sterile polypropylene tube. Each swab was approximately 5 mm in diameter and had a 13.3 cm plastic shaft to allow for precise sampling. As the oral microbiome has previously been shown to differ taxonomically among microhabitats [2], six different sample locations with varying surface structures were used to assess the absolute microbial abundance dynamics. Swab samples were always collected from the left posterior buccal vestibule, the middle of the back of the tongue, the occlusal surface of the terminal two molars on the right side of the mandible, the gingival margin of the last proximal molar on the right side of the mandible, the middle of the palate and the middle of the tip of the tongue (Figure 1). These samples will be referred to in this article as the posterior buccal vestibule, back of tongue, molars, gingiva, palate and tip of tongue respectively. These samples were taken just before lights out between 8.30–9.00pm, referred to as before sleep, and immediately on waking the following day between 6.00–6.30am, referred to as after sleep. Each swab was rotated clockwise 6 times at each location for sample collection. All participants’ samples were collected by the same researcher at the same locations before and after sleep using the same sampling technique described. Once the swab samples were collected, they were immediately placed back into the polypropylene tube and stored at −80°C until flow cytometric analysis.

### Sample preparation

Swab samples were thawed at room temperature immediately prior to sample preparation. Once thawed, swab tips were cut off into 1 ml of sterile (0.2 µm filtered and UV treated) TE buffer (10 mM Tris, 1 mM EDTA, pH 7.4, Sigma). Samples were then vortexed for 3 minutes to elute the bacteria and viruses from the swab tip.

Eluted swab samples were diluted (1:100) in 0.2 µm filtered TE buffer for optimal visualisation of bacterial and virus-like particle (VLP) populations. Diluted samples were then stained with SYBR-I Green (1:20,000 final dilution; Molecular Probes) and incubated for 10 minutes in the dark at 80°C as per previously established and optimised methods [17,18,21]. Control samples of sterile rayon swabs eluted in sterile TE buffer were prepared in the same manner as the participant swab samples. These samples were used to eliminate any background artefacts introduced during sample preparation or from the rayon swabs themselves (S1 Fig). Triplicates of each swab sample were prepared for analysis (S1-S4 Tables). Fluorescent beads (1 µm, Molecular Probes) were added to each sample at a concentration of 10^5^ beads ml^−1^ [22]. Using the bead fluorescence and concentration as a control, flow cytometric parameters were normalised [22].

### Flow cytometric analysis

**Figure 2.**
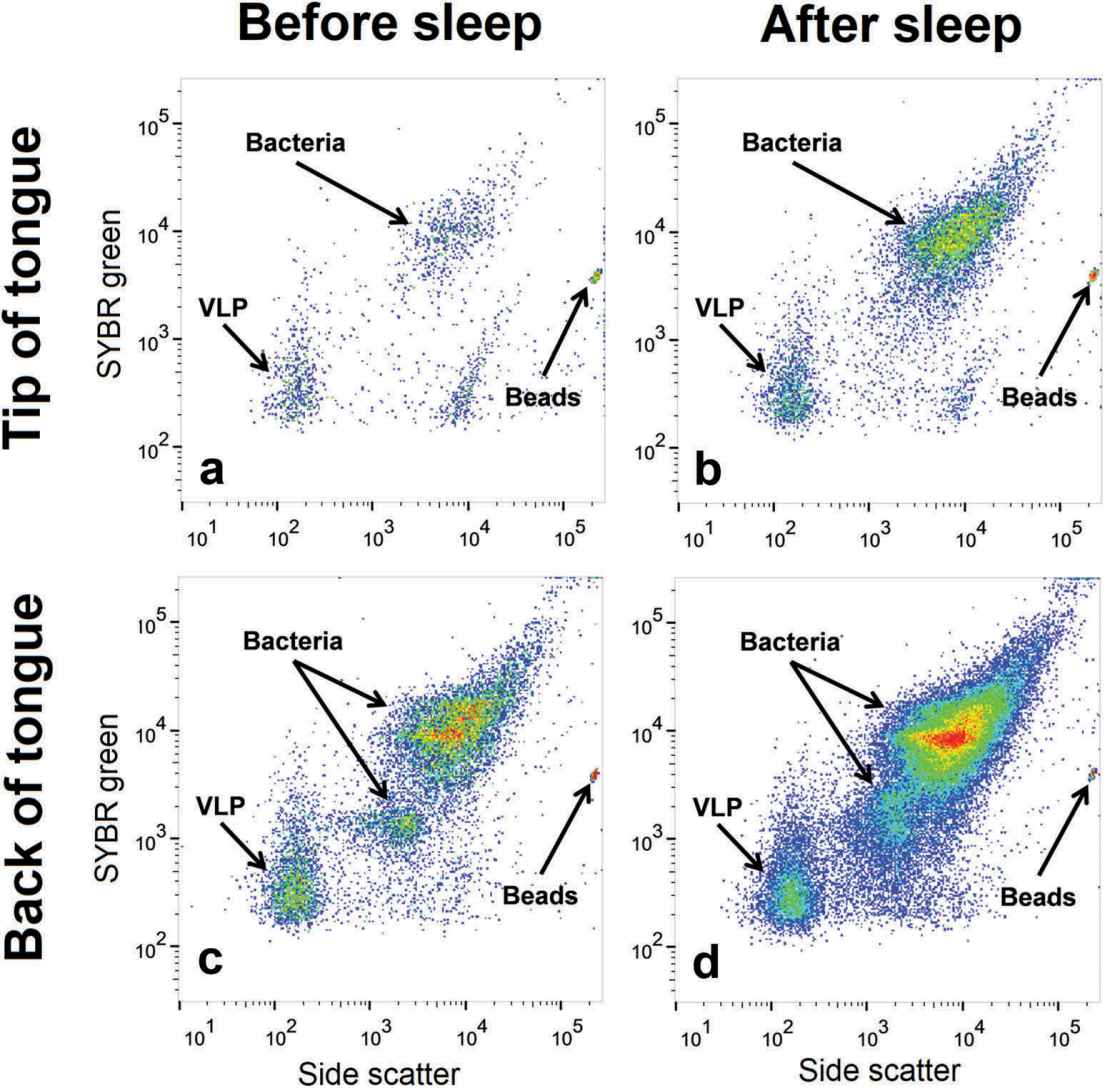
**Flow cytometric identification of bacterial and virus-like particle (VLP) populations**. Representative cytograms from one participant showing the bacterial and VLP populations at (a) the tip of the tongue before sleep (b) the tip of the tongue after sleep (c) the back of the tongue before sleep and (d) the back of the tongue after sleep. Bacterial and VLP abundances increased after sleep. Differences in bacterial and VLP abundances can also be seen between both sample locations

Bacterial and VLP populations from the oral swab samples were identified and enumerated using a FACSCanto II flow cytometer (Becton Dickinson). In this study, VLPs refer to small particles with a low DNA content, a characteristic of viruses. Green fluorescence, forward and side angle light scatter were recorded for all samples. SYBR green fluorescence was used to indicate the relative DNA content of a particle. Forward and side angle light scatter were used to differentiate these particles based on their relative size and granularity. As bacterial particles are larger and have more DNA than viral particles, they sit towards the top right of the cytogram. VLPs are smaller and have less DNA and are therefore situated towards the bottom left of the cytogram (Figure 2). Cell debris and macromolecules will be below the set threshold and are excluded from the analysis. Similarly, the scarcity and size of eukaryotes will be above the set threshold and excluded from the analysis. Phosphate-buffered saline was used as sheath fluid for the duration of the study.

Bacterial and VLP populations were analysed and enumerated using FlowJo software (Tree Star, Inc). SYBR green fluorescence and side scatter were used to differentiate between bacterial and VLP populations [16,17,23]. For consistency among participants, one bacterial population and one VLP population were compared and analysed (Figure 2).

### Data analysis

To examine microhabitat variation, an average abundance for each participant was taken, and then all the participants were averaged. Using the participants averages, the standard error of the mean (±SEM) was calculated for each location before and after sleep for bacteria and VLP. Large variations in the microbiomes among individuals is not uncommon in human studies [9,31–33]. Mann-Whitney U and Wilcoxon sign rank tests were run on the average participant bacterial and VLP abundances using the program MATLAB (MathWorks, Natick, Massachusetts, USA). MATLAB was also used to test for linear correlations between age and abundance for both raw and log transformed data. All p values calculated were corrected using the multiple comparisons hypothesis for false discovery [34]. Statistical significance was considered when p < 0.05. Cytoscape (version 3.5.1, http://www.cytoscape.org/) was used to create and visualise p < 0.05 filtered Pearson correlation coefficient networks for bacteria and VLPs both before and after sleep [35].

## Results

### Flow cytometric analysis

Bacterial and VLP populations were present in all sampled locations before and after sleep (an example is shown in Figure 2). For most participants, there was an increase in bacterial and VLP populations after sleep (Figure 2). Average abundances for bacteria and VLP populations at each oral microhabitat before and after sleep can be found in Tables 1 and 2. No significant linear correlations were observed between age and the abundance of bacteria or VLP (p > 0.05).

**Table 1.** **Mean (±SEM) bacterial abundances within the paediatric oral cavity before and after sleep compared using Wilcoxon sign rank test**. Error represents the standard error of the mean (SEM). Wilcoxon sign rank tests p values were corrected for false discovery rates

Sample location	Bacteria before sleep (± SEM)	Bacteria after sleep (± SEM)	Average increase in bacteria (± SEM)	p value
Posterior buccal vestibule	3.3 x 10^6^ (1.2 x 10^6^)	2.1 x 10^7^ (7.0 x 10^6^)	1.8 x 10^7^ (6.3 x 10^6^)	0.0025
Back of tongue	2.9 x 10^7^ (7.6 x 10^6^)	1.3 x 10^8^ (2.0 x 10^7^)	1.1 x 10^8^ (2.2 x 10^7^)	0.0032
Gingiva	1.7 x 10^7^ (7.7 x 10^6^)	4.1 x 10^7^ (7.6 x 10^6^)	2.3 x 10^7^ (8.6 x 10^6^)	0.0056
Palate	7.2 x 10^5^ (2.8 x 10^5^)	4.3 x 10^6^ (1.5 x 10^6^)	3.6 x 10^6^ (1.4 x 10^6^)	0.0013
Molars	4.8 x 10^6^ (1.6 x 10^6^)	1.9 x 10^7^ (5.4 x 10^6^)	1.4 x 10^7^ (5.6 x 10^6^)	0.0012
Tip of tongue	2.6 x 10^6^ (1.7 x 10^6^)	1.3 x 10^7^ (2.8 x 10^6^)	1.0 x 10^7^ (3.1 x 10^6^)	0.0016

**Table 2.** **Mean VLP abundances within the paediatric oral cavity before and after sleep compared using Wilcoxon sign rank test**. Error represents the standard error of the mean (SEM). Wilcoxon sign rank tests p values were corrected for false discovery rates

Sample location	VLP before sleep (± SEM)	VLP after sleep (± SEM)	Average increase in VLP (± SEM)	p value
Posterior buccal vestibule	5.7 x 10^6^ (1.9 x 10^6^)	5.7 x 10^7^ (2.3 x 10^7^)	5.1 x 10^7^ (2.2 x 10^7^)	0.00025
Back of tongue	2.2 x 10^7^ (6.3 x 10^6^)	9.2 x 10^7^ (5.0 x 10^7^)	7.0 x 10^7^ (4.7 x 10^7^)	0.00018
Gingiva	2.4 x 10^7^ (9.5 x 10^6^)	9.2 x 10^7^ (2.9 x 10^7^)	6.8 x 10^7^ (2.8 x 10^7^)	0.00021
Palate	1.9 x 10^6^ (1.0 x 10^6^)	1.4 x 10^7^ (9.9 x 10^6^)	1.2 x 10^7^ (9.7 x 10^6^)	0.000025
Molars	9.0 x 10^6^ (2.6 x 10^6^)	5.7 x 10^7^ (3.3 x 10^7^)	4.8 x 10^7^ (3.1 x 10^7^)	0.00016
Tip of tongue	3.6 x 10^6^ (1.2 x 10^6^)	1.7 x 10^7^ (8.2 x 10^6^)	1.4 x 10^7^ (8.4 x 10^6^)	0.00020

### Oral cavity bacterial abundance heterogeneity before sleep

**Figure 3.**
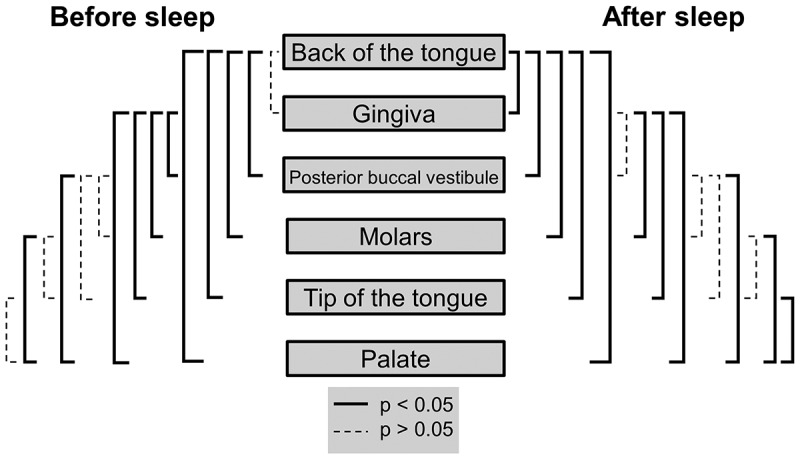
**Mann-Whitney U tests for bacterial heterogeneity in the oral cavity before and after sleep**. Significant differences between locations are represented by the solid black lines (p < 0.05). Non-significant differences are represented by the dashed black lines (p > 0.05). Mann-Whitney U test comparisons have been corrected for false discovery rates

No significant difference in bacterial abundances were detected between the back of the tongue and gingiva before sleep (p > 0.05) (Table 1; Figure 3). These two areas had the highest average bacterial counts, with participant averages ranging from 5.3 × 10^5^ (gingiva) to 8.7 × 10^7^ (back of tongue) bacteria (S5 Table). Both the back of the tongue and gingiva were found to have bacterial abundances significantly higher than the posterior buccal vestibule (p = 0.0018 and p = 0.016), palate (p = 0.0015 and p = 0.0012), molars (p = 0.0021 and p = 0.031) and tip of the tongue (p = 0.0012 and 0.0062) respectively (Table 1; Figure 3). The palate was the area in the mouth with the lowest bacterial abundances before sleep with participant’s averages ranging from 8.4 × 10^4^ to 2.2 × 10^6^ bacteria (Table 1; S5 Table). Although not significantly different to the tip of the tongue (p > 0.05), the palate was found to be significantly lower in bacterial abundance than the molars (p = 0.0059) and the posterior buccal vestibule (p = 0.0068) (Table 1; Figure 3). All other locations when compared to one another were not significantly different (p > 0.05) (Figure 3). Overall, there was approximately a 2.8 × 10^7^ abundance difference between the locations with the highest and lowest bacterial counts before sleep.

### Oral cavity bacterial abundance heterogeneity after sleep

The back of the tongue was the location with the highest bacterial abundances after sleep with participant’s averages ranging from 4.0 × 10^7^ to 2.1 × 10^8^ bacteria (Table 1; S6 Table). The back of the tongue also had significantly higher bacterial abundances after sleep when compared to all other locations (posterior buccal vestibule p = 0.00088; gingiva p = 0.0028; palate p = 0.0018; molars p = 0.00061 and tip of tongue p = 0.00091) (Table 1; Figure 3). The gingiva had significantly higher bacterial abundances after sleep than the molars (p = 0.016), the tip of the tongue (p = 0.0051) and the palate (p = 0.00061) (Table 1; Figure 3). The palate was the location with significantly lower counts of bacteria after sleep with participant averages ranging from 3.6 × 10^5^ to 1.4 × 10^7^ bacteria (Table 1; S6 Table). The palate was again significantly lower in abundance than the posterior buccal vestibule (p = 0.019), molars (p = 0.0072) and the tip of the tongue (p = 0.014) (Table 1; Figure 3). All other paired comparisons between locations were not significantly different (p > 0.05) (Figure 3). Overall, there was approximately a 1.3 × 10^8^ range in bacteria after sleep.

### Oral cavity VLP abundance heterogeneity before sleep

**Figure 4.**
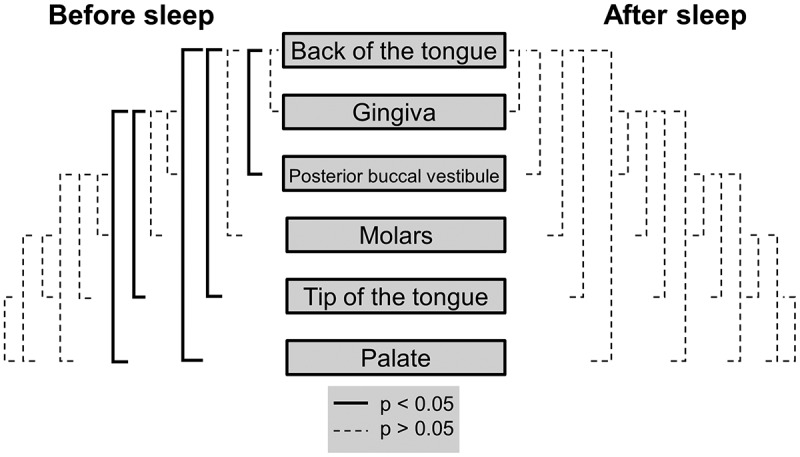
**Mann-Whitney U tests for VLP heterogeneity in the oral cavity before and after sleep**. Significant differences between locations are represented by the solid black lines (p < 0.05). Non-significant differences are represented by the dashed black lines (p > 0.05). Mann-Whitney U test comparisons have been corrected for false discovery rates

Less heterogeneity was observed between VLP abundances in the oral cavity before sleep than bacteria before sleep. The back of the tongue was found to be significantly higher in VLPs than the posterior buccal vestibule (p = 0.037), tip of the tongue (p = 0.016) and the palate (p = 0.0087; Table 2; Figure 4). The gingiva were also significantly higher in VLP abundance than the tip of the tongue (p = 0.019) and the palate (p = 0.013; Table 2; Figure 4). All other paired comparisons between oral sites did not show a significant difference (p > 0.05; Figure 4). Overall there was a range of 2.2 × 10^7^ VLPs before sleep.

### Oral cavity VLP abundance homogeneity after sleep

Homogeneity was observed among the average abundances of VLPs at each sampled location in the oral cavity after sleep (p > 0.05) (Figure 4). The average VLP abundances after sleep ranged from 1.4 ± 1.0 × 10^7^ at the palate to 9.2 ± 5.0 × 10^7^ at the back of the tongue (Table 2). Therefore, the average VLP abundance for all locations after sleep was 5.5 ± 1.2 × 10^7^.

### Bacterial and VLP increases during sleep

**Figure 5.**
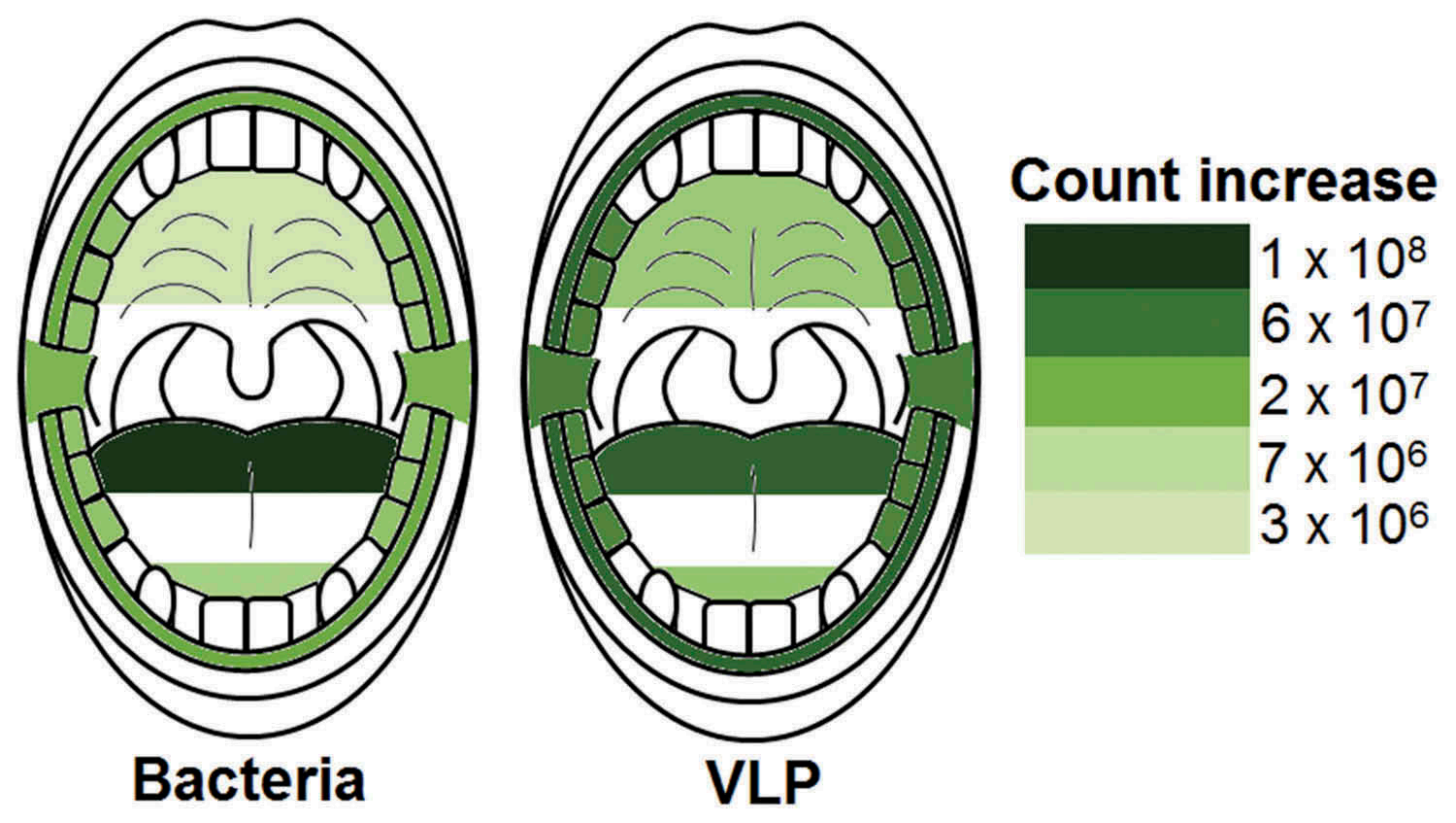
**Heat maps showing the average increase in bacteria and VLP after sleep at sampled locations**. All sampled oral locations significantly increased in bacteria and VLP during sleep (p < 0.05). The back of the tongue was the location that increased the most in both bacteria and VLPs during sleep with counts of 1.1 ± 0.2 × 10^8^ and 7.0 ± 4.7 × 10^7^ respectively. The palate increased the least in both bacteria and VLP with counts of 3.6 ± 1.4 × 10^6^ and 1.2 ± 1.0 × 10^7^ respectively

Corrected Wilcoxon sign rank test p values revealed that all sampled locations in the oral cavity significantly increased in bacterial and VLP abundances after sleep (p < 0.05; Tables 1 and 2; Figure 5). The palate was the location with the lowest average increase in microorganisms during sleep with 3.6 ± 1.4 × 10^6^ bacteria and 1.2 ± 1.0 × 10^7^ VLP (p < 0.002; Tables 1 and 2; Figure 5). The largest increase in bacteria and VLPs were observed at the back of the tongue with overall average count increases of 1.1 ± 0.2 × 10^8^ bacteria and 7.0 ± 4.7 × 10^7^ VLP (p < 0.004; Tables 1 and 2; Figure 5).

**Figure 6.**
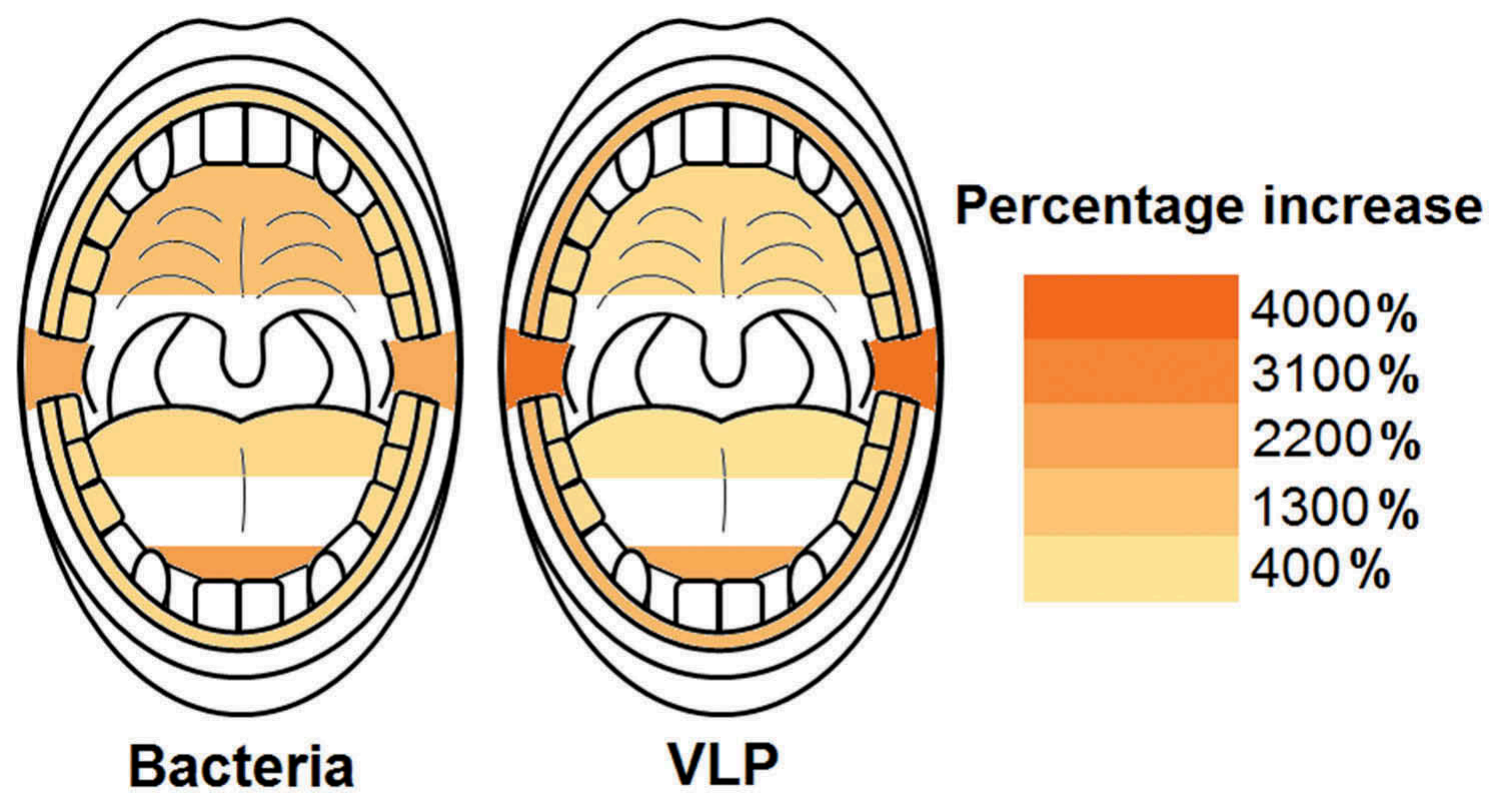
**Heat maps showing the average percentage increase in bacteria and VLP after sleep at sampled locations**. The molars, back of the tongue and gingiva were the locations with the lowest bacterial percentage increase during sleep (714%, 764% and 784% respectively). Likewise, the back of the tongue also had the lowest VLP percentage increase (416%). The tip of the tongue was the area with the highest bacterial percentage increase (2391%) and the posterior buccal vestibule the highest VLP percentage increase (3638%)

The largest percentage increases in bacteria were reported at the tip of the tongue and the posterior buccal vestibule with 2400% and 2100% increases respectively (Figure 6; Table S9). The gingiva, back of tongue and molars had the lowest percentage increases with 780%, 760% and 710% respectively (Figure 6; Table S9). A similar trend could be observed with the percentage increases for VLPs with the posterior buccal vestibule and tip of tongue having the highest increases with 3600% and 2100% respectively (Figure 6; Table S9). The back of the tongue had the lowest VLP percentage increase with 420% (Figure 6; Table S9).

### Bacterial and VLP network analysis

**Figure 7.**
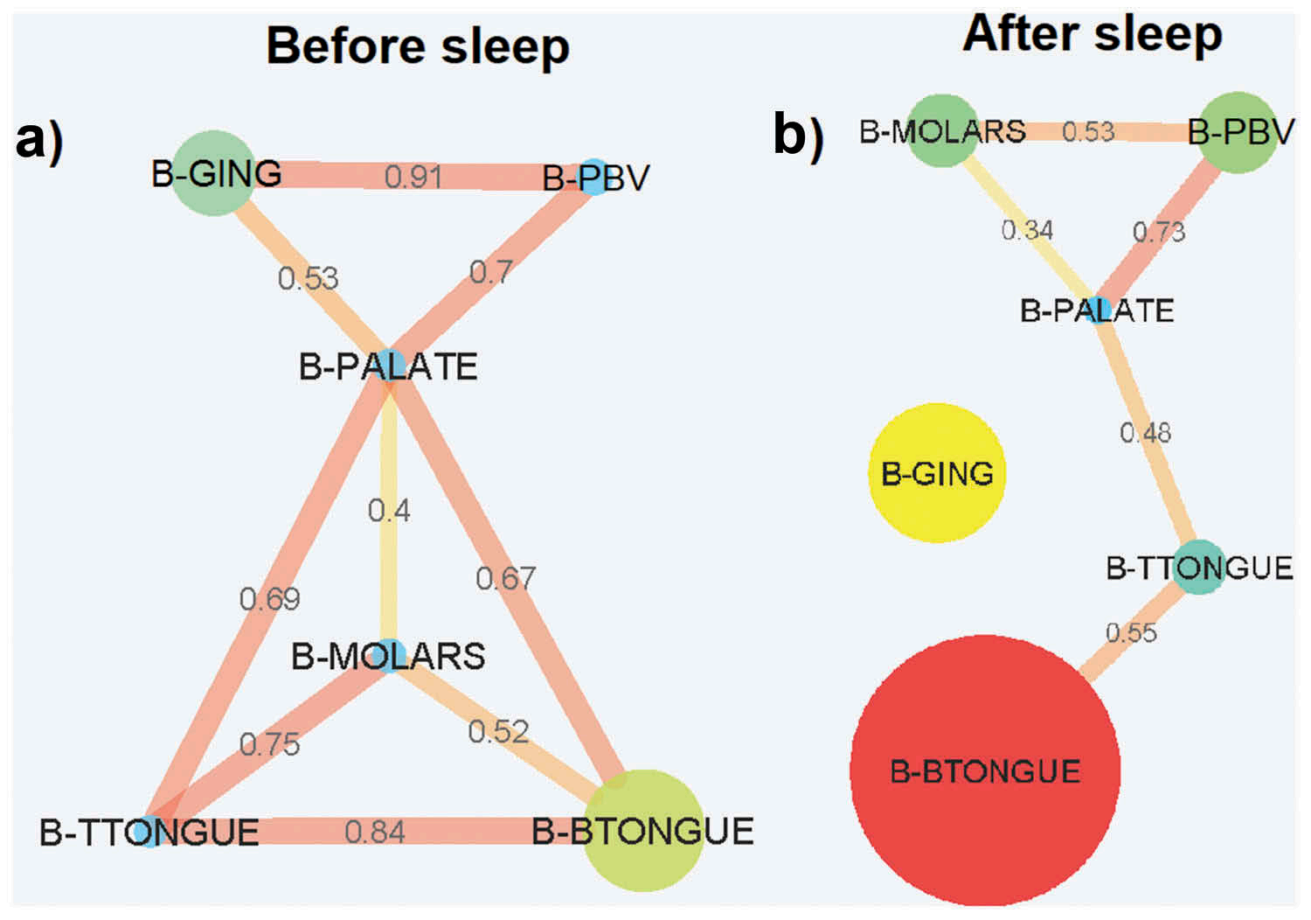
**Pearson correlation coefficient bacterial abundance networks (p < 0.05 filtered)**. Networks show the Pearson correlations between bacterial abundances for all samples locations (a) before sleep and (b) after sleep. Each node (circles) represents a sample location within the paediatric oral cavity. Node size is proportional to bacterial abundance at that location. The edges (lines) connecting each node represent a significant Pearson correlation (p < 0.05) in bacterial abundances between oral sites. The thickness of the edge relates to the strength of the Pearson correlation coefficient. B-GING = bacteria at the gingiva, B-PBV = bacteria at the posterior buccal vestibule, B-PALATE = bacteria at the palate, B-MOLARS = bacteria at the molars, B-TTONGUE = bacteria at the tip of the tongue and B-BTONGUE = bacteria at the back of the tongue

Pearson correlation coefficient bacterial abundance interaction network analysis before sleep revealed 9 connections between the 6 sampled locations, the strongest being between the gingiva and the posterior buccal vestibule (0.91) (Figure 7(a)). The palate was the only node connected to all other sample sites before sleep (Figure 7(a)). After sleep, the bacterial network breaks down with the gingiva no longer part of the abundance network (Figure 7(b)). The 5 connections seen in the network are no longer as strongly correlated compared to before sleep (Figure 7(b)). However, the correlation between the posterior buccal vestibule and the palate remains as strong as before. A new correlation is also formed between the molars and the posterior buccal vestibule (0.53) (Figure 7(b)). The palate is again the location with the greatest number of connections, this time with only 3. The strongest correlation in bacterial abundance after sleep was between the palate and the posterior buccal vestibule (0.73) (Figure 7(b)).

**Figure 8.**
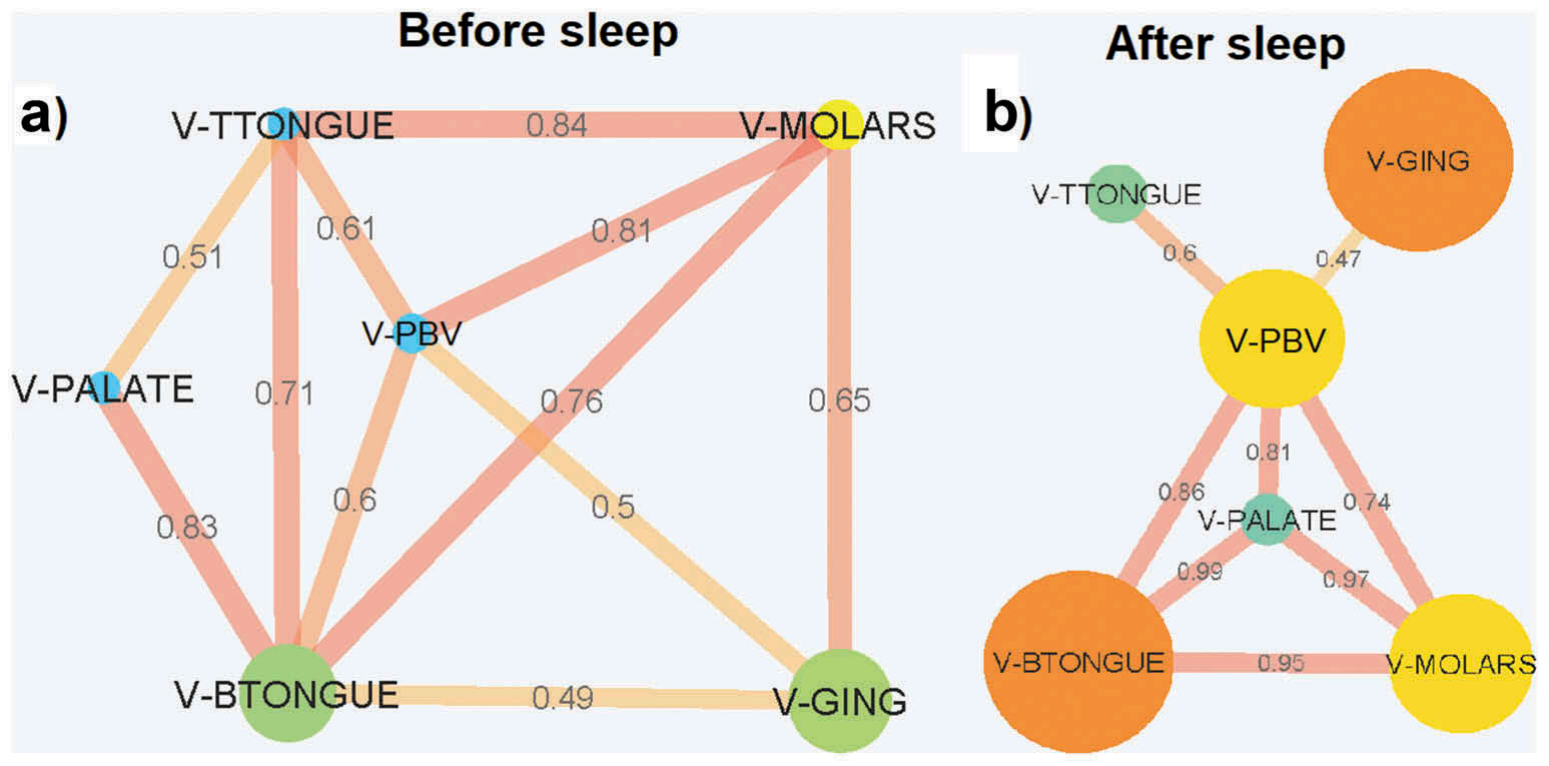
**Pearson correlation coefficient VLP abundance networks (p < 0.05 filtered)**. Networks show the Pearson correlations between VLP abundances for all samples locations (a) before sleep and (b) after sleep. Each node (circles) represents a sample location within the paediatric oral cavity. Node size is proportional to VLP abundance at that location. The edges (lines) connecting each node represent a significant Pearson correlation (p < 0.05) in VLP abundances between oral sites. The thickness of the edge relates to the strength of the Pearson correlation coefficient. V-GING = VLP at the gingiva, V-PBV = VLP at the posterior buccal vestibule, V-PALATE = VLP at the palate, V-MOLARS = VLP at the molars, V-TTONGUE = VLP at the tip of the tongue and V-BTONGUE = VLP at the back of the tongue

Eleven connections were observed in the Pearson correlation coefficient VLP abundance network before sleep, 5 of which were from all locations connecting with the back of the tongue (Figure 8(a)). The strongest correlation was between the molars and the tip of the tongue (0.84). Like with bacteria, the VLP network after sleep also broke down with fewer connections observed (Figure 8(b)). However, most of the correlations after sleep that are present remained strong. The correlations the back of the tongue has with the molars, palate and posterior buccal vestibule increased in strength after sleep (Figure 8(b)). The palate formed new strong correlations between the molars and the posterior buccal vestibule (Figure 8(b)). Of the 8 connections after sleep, the correlation between VLP abundances at the palate and the back of the tongue was the strongest (0.99). The gingiva and the posterior buccal vestibule were the areas with the weakest connection in the network (0.47; Figure 8(b)). All locations after sleep were correlated to the posterior buccal vestibule.

## Discussion

This study demonstrates that the microhabitats within the paediatric oral cavity significantly differ in absolute bacterial and VLP abundances (Figures 1, 3 and 4). Although many 16S taxonomic studies propose that the oral microbiome is relatively stable over longer periods of time [5–9], we demonstrate using flow cytometry (Figure 2), that during sleep the oral microbiome is highly dynamic and can significantly increase by counts of up to 100 million (Figures 5 and 6). Numerical changes add to previous taxonomic studies that suggest microhabitats in the oral cavity are influenced and structured by their immediate environment and hosts factors [36]. More importantly, our data highlights the need to control for sample location and time when conducting microbial abundance studies in the future.

Lower bacterial and VLP counts were observed at the palate compared to other microhabitats in the oral cavity (Tables 1 and 2). Due to the increased friction caused by the rough surface of the tongue, we speculate that the palate has a higher rate of mucosal shedding compared to other locations in the oral cavity. When these epithelial cells shed into the saliva, it brings with it the microbial rich mucous membrane lining the surface [2,37]. Therefore, there is less time between mucosal shedding events for a complex and abundant microbial community on this surface. This makes it a good model for the early development of mouth microbial communities.

Previous topographic tongue studies have shown that the highest colony-forming units for bacteria were produced from the area posterior to the circumvallate papillae [38]. This supports our finding of higher bacterial counts at the back of the tongue (Table 1; Figure 3). The papillae structures on the surface of the tongue provide an environment with a large surface area that encourages microbial growth. The crevasses and fissures formed by these structures trap small food particles and provide refuge for microbes from saliva flow and clearance [38].

Unlike bacteria, VLP abundances were numerically homogeneous after sleep (Figure 3; Table 2). Based on previous flow cytometric studies, these VLP populations are thought to reflect the viral abundance of an environment [17]. Although the reason for the VLP homogeneity could not be determined, it warrants further investigations into the oral microbial dynamics and what, if any, factor is controlling viral dispersal in the oral cavity during sleep [39,40].

Recently it has been shown that salivary flow creates gradients that influence the spatial organisation of the oral microbial communities [14]. Saliva production and flow rate is elevated during the day compared to night during sleep [11,12,41,42]. Food consumption and speech during the day leads to an increase in saliva production [3,41,43]. When deglutition occurs to clear food or excess saliva, the mucosal epithelial cells lining the oral cavity shed into the saliva taking with it the microbial rich mucosal layer [2,37]. Similarly, small plaque biofilm fragments break off and shed into the saliva. Therefore, during sleep when there is reduced saliva production and deglutition it would be expected that less microbial shedding would occur allowing more time for bacterial and VLP microbial community development. This supports our finding of a significant increases in bacterial and VLPs for all locations during sleep (p < 0.05; Figures 4 and 5; Tables 1 and 2). Mucin molecules in saliva are also believed to play a role in controlling the microbial communities in the oral cavity by promoting the aggregation and removal of oral bacteria [11,44–46]. Therefore, it could be postulated that when there is a reduction in saliva secretion during sleep [11,12,41,42], antimicrobial mucins will be at a lower concentration and will therefore result in an increase in oral bacteria.

Saliva is also involved in the dilution of sugars and buffering acids derived from both microbes and dietary intake [11,47]. With reduced ‘flushing’ of saliva through the oral cavity during sleep, it could be speculated that each microbial habitat in the oral cavity becomes more distinct and individualised based on its environment (i.e. pH or nutrient concentrations). This is supported by the breakdown of the bacterial network during sleep (Figure 7). This shows that there are fewer interactions between locations with each area acting more independently. However, although there is also a breakdown in the number of connections between locations for VLP during sleep, the strength of most of the correlations increases (Figure 8). This suggests that unlike bacteria, the VLP in the oral cavity are less likely to be impacted by the individualised conditions of each oral environment. This is again supported by the homogenisation of VLP abundances after sleep (Figure 4).

For this study, the participants involved did not engage in oral hygiene practices before sleep. The reasoning for this was to control for any biases introduced through individuality in the cleaning process. Therefore, it is expected that the abundance profiles generated in this study are on the higher end of the spectrum as plaque and nutrients in the form of trapped food particles that would have typically been removed remained. This could provide another possible explanation for the significant increases in microbial abundances.

The microbial abundances presented in this study are ones loosely associated with surfaces that can easily be removed by a swab. Recently, it has been suggested that the oral cavity may act as a reservoir for pathobionts involved in intestinal inflammatory diseases [48,49]. In addition, numerous studies have reported high relative abundances of microbes from oral origin within the gut of patients with liver cirrhosis, Crohn’s disease and colon cancer [50–52]. This suggests that it is important to monitor the microbial dynamics of the loosely associated microbial communities in the oral cavity as it may be important in understanding the microbial dynamics between the oral cavity and the digestive system in the future.

The results of this study demonstrate that during sleep, the paediatric oral microbiome in healthy sleepers is dynamic. Recently, Xu et al. have shown that the oral bacterial taxonomy in Obstructive Sleep Apnoea (OSA) is significantly perturbed in paediatric patients [53]. In addition, Wu et al. have also shown that changes in the nasal microbiome of adults is associated with severe OSA [54]. Whether these differences are also reflected in the absolute microbial abundance distributions within the oral cavity between healthy sleepers and people experiencing ongoing disturbed sleep (e.g. shift workers, insomnia) is unknown. Studies now suggest that paediatric sleep disorder breathing is associated with increased blood pressure and heart rate [55–58]. In addition, hypertension in childhood is reported to be predictive of hypertension in adulthood [59]. Given the relationship between sleep disorders, cardiovascular disease and changes in microbiome, future studies should investigate whether this growth pattern is also altered in these pathological states.

## Conclusions

In conclusion, our results demonstrate that flow cytometry can be used as a tool to enumerate bacteria and VLPs from oral swab samples. Here we show that the oral cavity is an active microbial environment during sleep and that changes in oral environmental conditions could have a large impact on the absolute microbial abundances observed. Microbial abundance heterogeneity then means that some microhabitats would contribute disproportionately to the overall microbial abundance of saliva. This highlights the importance of defining the oral cavity by its various microhabitats and controlling for sample collection time in future microbial abundance studies. The large ranges observed among healthy individual’s oral cavities (S5-S8 Tables) could indicate that high microbial abundances may not be indicative of oral related illnesses. This suggests, like taxonomy [31,60], microbial abundances are distinct to individuals even at the paediatric age group, where there has been less time for community and abundance divergence. As the oral microbiome taxonomically changes with each developmental stage of life [2,61], future studies into the absolute microbial counts at different age groups will assist in identifying if these microbial abundance dynamics are specific to age.

## Supplementary Material

Supplemental Material

## Declarations

Ethics was approved by the Human Research Ethics Committees of the Women’s and Children’s Hospital and the University of Adelaide, South Australia (REC2495\3\16(HREC\12\WCHN\52;SSA\12\WCH\53). The study has been conducted in accordance with the 1964 Declaration of Helsinki and its later amendments. Parents of participants provided written consent and children written assent for involvement in the study.
