# Supplementary material for: The microbial abundance dynamics of the paediatric oral cavity before and after sleep
Source: J Oral Microbiol. 2020 Mar 30;12(1):1741254. doi: 10.1080/20002297.2020.1741254 (PMC7170375; doi:10.1080/20002297.2020.1741254)
Supplement: Supplemental Material [file ZJOM_A_1741254_SM8966.zip › Supplementary/S1_4_Tables.docx]

**S1 Table. Bacterial abundances in the oral cavity before sleep for each participant. Eluted swab samples were prepared and run three times on the flow cytometer as a methods replicate (R). Participant’s average bacterial abundances before sleep were calculated using these three values (S5 Table).**

|  | | **Participant** | | | | | | | | | |
| --- | --- | --- | --- | --- | --- | --- | --- | --- | --- | --- | --- |
|  |  | **1** | **2** | **3** | **4** | **5** | **6** | **7** | **8** | **9** | **10** |
| **Posterior buccal vestibule** | **R1** | 1.6E+07 | 4.4E+06 | 4.2E+06 | 3.7E+06 | 3.7E+06 | 2.3E+05 | 4.7E+05 | 5.4E+05 | 1.2E+06 | 2.3E+06 |
|  | **R2** | 1.0E+07 | 4.2E+06 | 4.5E+06 | 3.4E+06 | 3.8E+06 | 2.5E+05 | 4.5E+05 | 6.8E+05 | 1.4E+06 | 2.3E+06 |
|  | **R3** | 1.2E+07 | 3.9E+06 | 4.1E+06 | 3.9E+06 | 1.2E+06 | 2.2E+05 | 5.8E+05 | 7.0E+05 | 7.2E+05 | 2.1E+06 |
| **Back of tongue** | **R1** | 2.6E+07 | 8.9E+07 | 3.3E+07 | 4.6E+07 | 9.6E+06 | 2.4E+07 | 3.9E+07 | 1.6E+07 | 9.4E+06 | 8.1E+06 |
|  | **R2** | 2.3E+07 | 9.8E+07 | 2.9E+07 | 4.6E+07 | 5.8E+06 | 2.6E+07 | 3.6E+07 | 1.9E+07 | 9.2E+06 | 6.9E+06 |
|  | **R3** | 2.2E+07 | 7.3E+07 | 2.5E+07 | 4.6E+07 | 8.8E+06 | 2.8E+07 | 3.9E+07 | 1.3E+07 | 8.0E+06 | 7.6E+06 |
| **Gingivae** | **R1** | 6.8E+07 | 1.4E+07 | 2.2E+07 | 3.8E+06 | 1.1E+07 | 6.2E+05 | 1.4E+07 | 1.1E+07 | 1.4E+07 | 4.7E+06 |
|  | **R2** | 8.9E+07 | 1.8E+07 | 1.8E+07 | 2.8E+06 | 7.4E+06 | 4.5E+05 | 1.6E+07 | 1.3E+07 | 1.2E+07 | 3.2E+06 |
|  | **R3** | 9.9E+07 | 1.7E+07 | 1.4E+07 | 3.3E+06 | 9.7E+06 | 5.0E+05 | 1.3E+07 | 9.1E+06 | 1.1E+07 | 4.5E+06 |
| **Palate** | **R1** | 2.1E+06 | 2.1E+06 | 2.3E+05 | 1.7E+06 | 1.9E+05 | 1.5E+05 | 7.3E+04 | 9.2E+04 | 4.4E+05 | 2.4E+05 |
|  | **R2** | 1.9E+06 | 2.3E+06 | 1.9E+05 | 1.6E+06 | 1.6E+05 | 1.6E+05 | 9.8E+04 | 7.5E+04 | 4.7E+05 | 2.5E+05 |
|  | **R3** | 2.1E+06 | 2.3E+06 | 1.2E+05 | 1.5E+06 | 8.2E+04 | 1.7E+05 | 8.9E+04 | 8.5E+04 | 3.7E+05 | 1.9E+05 |
| **Molars** | **R1** | 9.8E+05 | 1.7E+07 | 8.2E+06 | 3.7E+06 | 4.9E+06 | 5.3E+05 | 5.4E+05 | 3.8E+05 | 1.0E+07 | 3.9E+06 |
|  | **R2** | 1.9E+06 | 1.5E+07 | 7.7E+06 | 3.3E+06 | 4.5E+06 | 5.3E+05 | 6.4E+05 | 3.4E+05 | 9.8E+06 | 3.4E+06 |
|  | **R3** | 1.7E+06 | 1.6E+07 | 6.9E+06 | 2.9E+06 | 4.0E+06 | 4.6E+05 | 4.6E+05 | 2.0E+05 | 1.2E+07 | 3.1E+06 |
| **Tip of tongue** | **R1** | 2.3E+06 | 1.7E+07 | 4.6E+05 | 1.6E+06 | 2.3E+05 | 1.8E+05 | 5.4E+05 | 3.2E+05 | 1.2E+06 | 2.2E+06 |
|  | **R2** | 2.0E+06 | 1.9E+07 | 5.6E+05 | 1.6E+06 | 3.4E+05 | 1.9E+05 | 4.3E+05 | 2.2E+05 | 9.6E+05 | 1.3E+06 |
|  | **R3** | 3.0E+06 | 1.6E+07 | 5.0E+05 | 1.4E+06 | 1.1E+05 | 2.2E+05 | 3.8E+05 | 2.4E+05 | 9.7E+05 | 1.5E+06 |

**S2 Table. Bacterial abundances in the oral cavity after sleep for each participant. Eluted swab samples were prepared and run three times on the flow cytometer as a methods replicate (R). Participant’s average bacterial abundances after sleep were calculated using these three values (S6 Table).**

|  | | **Participant** | | | | | | | | | |
| --- | --- | --- | --- | --- | --- | --- | --- | --- | --- | --- | --- |
|  |  | **1** | **2** | **3** | **4** | **5** | **6** | **7** | **8** | **9** | **10** |
| **Posterior buccal vestibule** | **R1** | 8.1E+07 | 3.9E+06 | 2.5E+06 | 4.1E+07 | 7.1E+06 | 3.3E+07 | 6.8E+06 | 2.9E+06 | 1.2E+07 | 2.0E+07 |
|  | **R2** | 5.0E+07 | 3.3E+06 | 3.4E+06 | 5.1E+07 | 8.6E+06 | 3.9E+07 | 1.3E+07 | 2.9E+06 | 8.6E+06 | 3.3E+07 |
|  | **R3** | 7.4E+07 | 2.8E+06 | 2.9E+06 | 4.4E+07 | 8.4E+06 | 3.6E+07 | 9.4E+06 | 1.7E+06 | 1.2E+07 | 2.7E+07 |
| **Back of tongue** | **R1** | 1.4E+08 | 8.0E+07 | 1.9E+08 | 1.5E+08 | 8.9E+07 | 1.2E+08 | 4.0E+07 | 7.5E+07 | 2.3E+08 | 2.3E+08 |
|  | **R2** | 1.2E+08 | 1.6E+08 | 1.6E+08 | 2.4E+08 | 7.5E+07 | 7.6E+07 | 4.6E+07 | 5.7E+07 | 2.1E+08 | 2.3E+08 |
|  | **R3** | 1.6E+08 | 1.9E+08 | 1.7E+08 | 2.4E+08 | 4.8E+07 | 1.0E+08 | 3.4E+07 | 5.7E+07 | 1.7E+08 | 1.7E+08 |
| **Gingivae** | **R1** | 6.3E+07 | 5.5E+07 | 8.3E+06 | 1.8E+07 | 2.7E+07 | 2.3E+07 | 2.2E+07 | 7.9E+07 | 3.2E+07 | 7.5E+07 |
|  | **R2** | 6.5E+07 | 6.5E+07 | 2.4E+07 | 1.7E+07 | 3.4E+07 | 2.6E+07 | 1.8E+07 | 7.4E+07 | 4.5E+07 | 7.0E+07 |
|  | **R3** | 7.5E+07 | 3.1E+07 | 1.9E+07 | 1.7E+07 | 1.8E+07 | 2.3E+07 | 2.0E+07 | 5.1E+07 | 3.6E+07 | 9.3E+07 |
| **Palate** | **R1** | 9.6E+06 | 1.2E+06 | 2.2E+06 | 3.0E+06 | 2.4E+06 | 1.2E+07 | 4.9E+05 | 4.1E+05 | 4.3E+06 | 2.2E+06 |
|  | **R2** | 1.5E+07 | 2.2E+06 | 1.5E+06 | 4.8E+06 | 2.3E+06 | 1.5E+07 | 7.1E+05 | 3.3E+05 | 5.5E+06 | 1.8E+06 |
|  | **R3** | 9.9E+06 | 2.5E+06 | 1.6E+06 | 3.0E+06 | 3.3E+06 | 1.4E+07 | 6.2E+05 | 3.4E+05 | 4.6E+06 | 1.5E+06 |
| **Molars** | **R1** | 5.5E+07 | 1.1E+07 | 1.3E+07 | 1.3E+07 | 4.7E+07 | 2.6E+06 | 4.2E+06 | 3.3E+06 | 2.4E+07 | 1.6E+07 |
|  | **R2** | 4.9E+07 | 1.7E+07 | 1.7E+07 | 1.4E+07 | 4.2E+07 | 2.9E+06 | 3.4E+06 | 2.5E+06 | 2.4E+07 | 2.1E+07 |
|  | **R3** | 6.8E+07 | 1.6E+07 | 1.4E+07 | 2.0E+07 | 2.3E+07 | 2.6E+06 | 2.8E+06 | 2.0E+06 | 2.7E+07 | 1.5E+07 |
| **Tip of tongue** | **R1** | 9.2E+06 | 1.4E+07 | 5.4E+06 | 2.0E+07 | 1.0E+07 | 2.5E+07 | 1.1E+06 | 3.9E+06 | 2.7E+07 | 2.3E+07 |
|  | **R2** | 7.0E+06 | 1.4E+07 | 5.2E+06 | 1.6E+07 | 1.1E+07 | 2.6E+07 | 1.5E+06 | 4.5E+06 | 2.6E+07 | 1.6E+07 |
|  | **R3** | 6.5E+06 | 1.5E+07 | 4.6E+06 | 1.7E+07 | 6.6E+06 | 2.7E+07 | 9.3E+05 | 3.5E+06 | 2.4E+07 | 1.7E+07 |

**S3 Table. VLP abundances in the oral cavity before sleep for each participant. Eluted swab samples were prepared and run three times on the flow cytometer as a methods replicate (R). Participant’s average VLP abundances before sleep were calculated using these three values (S7 Table).**

|  | | **Participant** | | | | | | | | | |
| --- | --- | --- | --- | --- | --- | --- | --- | --- | --- | --- | --- |
|  |  | **1** | **2** | **3** | **4** | **5** | **6** | **7** | **8** | **9** | **10** |
| **Posterior buccal vestibule** | **R1** | 1.4E+07 | 8.2E+06 | 2.1E+06 | 6.0E+06 | 3.2E+06 | 2.5E+05 | 1.9E+06 | 1.2E+06 | 1.3E+06 | 1.5E+07 |
|  | **R2** | 1.4E+07 | 1.0E+07 | 2.7E+06 | 6.7E+06 | 4.1E+06 | 4.7E+05 | 1.5E+06 | 1.6E+06 | 1.2E+06 | 2.7E+07 |
|  | **R3** | 1.4E+07 | 8.9E+06 | 2.0E+06 | 5.3E+06 | 3.1E+06 | 4.1E+05 | 2.1E+06 | 1.3E+06 | 1.1E+06 | 9.2E+06 |
| **Back of tongue** | **R1** | 5.2E+07 | 3.3E+07 | 8.7E+06 | 7.6E+07 | 9.9E+06 | 3.9E+06 | 2.2E+06 | 1.0E+07 | 2.1E+07 | 2.5E+07 |
|  | **R2** | 4.4E+07 | 3.6E+07 | 7.5E+06 | 5.8E+07 | 8.8E+06 | 3.8E+06 | 1.1E+06 | 1.2E+07 | 2.0E+07 | 2.7E+07 |
|  | **R3** | 4.9E+07 | 2.6E+07 | 1.2E+07 | 4.9E+07 | 1.0E+07 | 4.2E+06 | 1.5E+06 | 6.3E+06 | 1.2E+07 | 2.9E+07 |
| **Gingivae** | **R1** | 8.9E+07 | 4.3E+07 | 1.6E+07 | 1.3E+07 | 1.1E+07 | 1.5E+06 | 1.7E+07 | 2.5E+07 | 4.9E+06 | 3.3E+06 |
|  | **R2** | 9.6E+07 | 4.9E+07 | 1.4E+07 | 1.4E+07 | 1.4E+07 | 1.3E+06 | 1.2E+07 | 2.4E+07 | 5.8E+06 | 2.8E+06 |
|  | **R3** | 1.2E+08 | 5.6E+07 | 2.2E+07 | 1.2E+07 | 8.4E+06 | 1.3E+06 | 1.7E+07 | 1.2E+07 | 4.8E+06 | 4.6E+06 |
| **Palate** | **R1** | 3.2E+06 | 1.6E+06 | 2.3E+05 | 9.1E+06 | 2.3E+05 | 1.4E+06 | 8.7E+04 | 4.9E+05 | 1.4E+06 | 8.4E+05 |
|  | **R2** | 2.4E+06 | 1.6E+06 | 8.0E+04 | 1.3E+07 | 6.1E+05 | 1.5E+06 | 9.0E+04 | 2.4E+05 | 8.9E+05 | 6.8E+05 |
|  | **R3** | 3.0E+06 | 2.1E+06 | 1.8E+05 | 1.1E+07 | 2.2E+05 | 1.2E+06 | 1.1E+05 | 1.9E+05 | 5.6E+05 | 4.6E+05 |
| **Molars** | **R1** | 1.9E+07 | 1.9E+07 | 1.2E+06 | 1.3E+07 | 3.4E+06 | 1.1E+06 | 9.2E+04 | 7.3E+06 | 7.8E+06 | 1.3E+07 |
|  | **R2** | 1.7E+07 | 2.3E+07 | 1.6E+06 | 9.1E+06 | 3.1E+06 | 1.2E+06 | 1.3E+05 | 7.9E+06 | 8.1E+06 | 2.1E+07 |
|  | **R3** | 2.4E+07 | 2.4E+07 | 1.1E+06 | 1.5E+07 | 2.0E+06 | 8.1E+05 | 8.6E+04 | 3.9E+06 | 7.7E+06 | 1.4E+07 |
| **Tip of tongue** | **R1** | 3.6E+06 | 1.0E+07 | 4.1E+05 | 9.1E+06 | 2.7E+05 | 5.5E+05 | 3.4E+05 | 2.2E+06 | 8.5E+05 | 6.4E+06 |
|  | **R2** | 4.0E+06 | 1.2E+07 | 6.3E+05 | 7.6E+06 | 8.1E+05 | 3.9E+05 | 1.5E+05 | 2.0E+06 | 8.5E+05 | 8.7E+06 |
|  | **R3** | 4.1E+06 | 1.2E+07 | 8.3E+05 | 7.9E+06 | 5.2E+05 | 4.0E+05 | 2.0E+05 | 4.7E+06 | 6.9E+05 | 4.6E+06 |

**S4 Table. VLP abundances in the oral cavity after sleep for each participant. Eluted swab samples were prepared and run three times on the flow cytometer as a methods replicate (R). Participant’s average VLP abundances after sleep were calculated using these three values (S8 Table).**

|  | | **Participant** | | | | | | | | | | | | | | | | | | |
| --- | --- | --- | --- | --- | --- | --- | --- | --- | --- | --- | --- | --- | --- | --- | --- | --- | --- | --- | --- | --- |
|  |  | **1** | | **2** | | **3** | | **4** | | **5** | | **6** | | **7** | | **8** | | **9** | | **10** |
| **Posterior buccal vestibule** | **R1** | 1.9E+08 | 2.4E+06 | | 9.5E+05 | | 7.4E+07 | | 1.7E+06 | | 1.1E+08 | | 4.0E+06 | | 8.2E+06 | | 7.9E+06 | | 1.3E+08 | |
|  | **R2** | 2.5E+08 | 7.6E+06 | | 1.0E+06 | | 1.4E+08 | | 3.4E+06 | | 1.1E+08 | | 2.4E+06 | | 7.9E+06 | | 1.3E+07 | | 1.0E+08 | |
|  | **R3** | 1.8E+08 | 6.4E+06 | | 8.4E+05 | | 1.0E+08 | | 4.0E+06 | | 1.4E+08 | | 2.9E+06 | | 3.1E+06 | | 1.1E+07 | | 9.6E+07 | |
| **Back of tongue** | **R1** | 3.9E+08 | 3.1E+07 | | 1.7E+07 | | 8.3E+07 | | 8.9E+06 | | 8.2E+07 | | 1.0E+07 | | 1.2E+07 | | 1.2E+07 | | 5.6E+07 | |
|  | **R2** | 4.6E+08 | 6.2E+07 | | 1.4E+07 | | 1.5E+08 | | 6.6E+06 | | 1.0E+08 | | 8.4E+06 | | 1.2E+07 | | 1.2E+07 | | 6.1E+07 | |
|  | **R3** | 7.3E+08 | 6.2E+07 | | 2.1E+07 | | 1.5E+08 | | 6.6E+06 | | 1.1E+08 | | 8.0E+06 | | 7.9E+06 | | 1.1E+07 | | 6.9E+07 | |
| **Gingivae** | **R1** | 1.7E+08 | 2.1E+08 | | 4.0E+06 | | 4.3E+07 | | 7.0E+06 | | 8.8E+07 | | 3.9E+06 | | 1.2E+08 | | 3.0E+07 | | 3.2E+08 | |
|  | **R2** | 1.1E+08 | 2.7E+08 | | 5.3E+06 | | 9.2E+07 | | 1.6E+07 | | 1.1E+08 | | 5.6E+06 | | 1.1E+08 | | 3.5E+07 | | 2.3E+08 | |
|  | **R3** | 1.6E+08 | 6.0E+07 | | 6.8E+06 | | 2.6E+07 | | 4.8E+06 | | 8.0E+07 | | 7.6E+06 | | 8.0E+07 | | 3.0E+07 | | 3.1E+08 | |
| **Palate** | **R1** | 8.7E+07 | 2.9E+06 | | 4.2E+05 | | 1.1E+07 | | 3.3E+06 | | 1.3E+07 | | 2.6E+05 | | 9.8E+05 | | 1.7E+06 | | 6.7E+06 | |
|  | **R2** | 1.6E+08 | 2.8E+06 | | 5.5E+05 | | 1.6E+07 | | 3.6E+06 | | 1.4E+07 | | 4.9E+05 | | 1.4E+06 | | 2.3E+06 | | 5.2E+06 | |
|  | **R3** | 6.3E+07 | 1.5E+06 | | 6.1E+05 | | 1.1E+07 | | 4.2E+06 | | 1.3E+07 | | 4.9E+05 | | 9.0E+05 | | 2.1E+06 | | 5.3E+06 | |
| **Molars** | **R1** | 2.6E+08 | 1.7E+07 | | 1.2E+07 | | 1.8E+07 | | 3.3E+07 | | 1.0E+07 | | 1.1E+06 | | 4.1E+06 | | 6.9E+07 | | 7.0E+07 | |
|  | **R2** | 2.4E+08 | 1.6E+07 | | 1.0E+07 | | 2.6E+07 | | 2.4E+07 | | 1.0E+07 | | 1.3E+06 | | 4.8E+06 | | 4.4E+07 | | 5.8E+07 | |
|  | **R3** | 5.4E+08 | 2.1E+07 | | 1.6E+07 | | 2.6E+07 | | 3.0E+07 | | 9.7E+06 | | 2.1E+06 | | 4.3E+06 | | 6.9E+07 | | 5.8E+07 | |
| **Tip of tongue** | **R1** | 3.6E+07 | 4.9E+06 | | 7.2E+05 | | 3.6E+07 | | 1.2E+06 | | 8.2E+07 | | 4.9E+05 | | 7.1E+06 | | 3.4E+06 | | 2.3E+07 | |
|  | **R2** | 1.8E+07 | 5.3E+06 | | 5.6E+05 | | 2.6E+07 | | 1.7E+06 | | 9.3E+07 | | 3.7E+05 | | 1.0E+07 | | 3.0E+06 | | 2.0E+07 | |
|  | **R3** | 1.7E+07 | 4.0E+06 | | 9.9E+05 | | 1.4E+07 | | 1.3E+06 | | 8.4E+07 | | 1.8E+05 | | 7.6E+06 | | 2.9E+06 | | 1.6E+07 | |
