# Supplementary material for: The microbial abundance dynamics of the paediatric oral cavity before and after sleep
Source: J Oral Microbiol. 2020 Mar 30;12(1):1741254. doi: 10.1080/20002297.2020.1741254 (PMC7170375; doi:10.1080/20002297.2020.1741254)
Supplement: Supplemental Material [file ZJOM_A_1741254_SM8966.zip › Supplementary/S5_9_Tables.docx]

**S5 Table. Participant’s average bacterial abundances for each sample site in the oral cavity before sleep.**

| **Participant** | **Posterior buccal vestibule** | **Back of tongue** | **Gingivae** | **Palate** | **Molars** | **Tip of tongue** |
| --- | --- | --- | --- | --- | --- | --- |
| 1 | 1.28E+07 | 2.38E+07 | 8.52E+07 | 2.05E+06 | 1.51E+06 | 2.45E+06 |
| 2 | 4.19E+06 | 8.66E+07 | 1.62E+07 | 2.22E+06 | 1.59E+07 | 1.74E+07 |
| 3 | 4.28E+06 | 2.91E+07 | 1.79E+07 | 1.80E+05 | 7.61E+06 | 5.05E+05 |
| 4 | 3.67E+06 | 4.60E+07 | 3.32E+06 | 1.63E+06 | 3.29E+06 | 1.57E+06 |
| 5 | 2.91E+06 | 8.05E+06 | 9.52E+06 | 1.44E+05 | 4.45E+06 | 2.29E+05 |
| 6 | 2.36E+05 | 2.61E+07 | 5.25E+05 | 1.61E+05 | 5.06E+05 | 1.99E+05 |
| 7 | 4.99E+05 | 3.82E+07 | 1.46E+07 | 8.65E+04 | 5.46E+05 | 4.50E+05 |
| 8 | 6.39E+05 | 1.62E+07 | 1.12E+07 | 8.41E+04 | 3.08E+05 | 2.64E+05 |
| 9 | 1.08E+06 | 8.85E+06 | 1.23E+07 | 4.29E+05 | 1.07E+07 | 1.03E+06 |
| 10 | 2.25E+06 | 7.53E+06 | 4.16E+06 | 2.27E+05 | 3.48E+06 | 1.64E+06 |

**S6 Table. Participant’s average bacterial abundances for each sample site in the oral cavity after sleep.**

| **Participant** | **Posterior buccal vestibule** | **Back of tongue** | **Gingivae** | **Palate** | **Molars** | **Tip of tongue** |
| --- | --- | --- | --- | --- | --- | --- |
| 1 | 6.83E+07 | 1.40E+08 | 6.78E+07 | 1.17E+07 | 5.73E+07 | 7.59E+06 |
| 2 | 3.36E+06 | 1.42E+08 | 5.00E+07 | 1.99E+06 | 1.44E+07 | 1.42E+07 |
| 3 | 2.94E+06 | 1.75E+08 | 1.69E+07 | 1.73E+06 | 1.48E+07 | 5.07E+06 |
| 4 | 4.50E+07 | 2.07E+08 | 1.75E+07 | 3.60E+06 | 1.58E+07 | 1.74E+07 |
| 5 | 8.05E+06 | 7.06E+07 | 2.60E+07 | 2.66E+06 | 3.72E+07 | 9.30E+06 |
| 6 | 3.61E+07 | 9.99E+07 | 2.41E+07 | 1.39E+07 | 2.69E+06 | 2.59E+07 |
| 7 | 9.73E+06 | 3.99E+07 | 1.99E+07 | 6.06E+05 | 3.47E+06 | 1.18E+06 |
| 8 | 2.49E+06 | 6.31E+07 | 6.81E+07 | 3.60E+05 | 2.62E+06 | 3.94E+06 |
| 9 | 1.09E+07 | 2.03E+08 | 3.78E+07 | 4.82E+06 | 2.51E+07 | 2.53E+07 |
| 10 | 2.68E+07 | 2.10E+08 | 7.97E+07 | 1.83E+06 | 1.74E+07 | 1.86E+07 |

**S7 Table. Participant’s average VLP abundances for each sample site in the oral cavity before sleep.**

| **Participant** | **Posterior buccal vestibule** | **Back of tongue** | **Gingivae** | **Palate** | **Molars** | **Tip of tongue** |
| --- | --- | --- | --- | --- | --- | --- |
| 1 | 1.41E+07 | 4.83E+07 | 1.00E+08 | 2.89E+06 | 2.04E+07 | 3.90E+06 |
| 2 | 9.14E+06 | 3.15E+07 | 4.93E+07 | 1.77E+06 | 2.23E+07 | 1.13E+07 |
| 3 | 2.23E+06 | 9.23E+06 | 1.73E+07 | 1.63E+05 | 1.33E+06 | 6.24E+05 |
| 4 | 5.97E+06 | 6.12E+07 | 1.27E+07 | 1.08E+07 | 1.24E+07 | 8.18E+06 |
| 5 | 3.49E+06 | 9.70E+06 | 1.11E+07 | 3.55E+05 | 2.82E+06 | 5.29E+05 |
| 6 | 3.76E+05 | 3.96E+06 | 1.37E+06 | 1.37E+06 | 1.02E+06 | 4.45E+05 |
| 7 | 1.82E+06 | 1.62E+06 | 1.52E+07 | 9.42E+04 | 1.04E+05 | 2.28E+05 |
| 8 | 1.40E+06 | 9.63E+06 | 2.01E+07 | 3.04E+05 | 6.39E+06 | 2.97E+06 |
| 9 | 1.19E+06 | 1.80E+07 | 5.19E+06 | 9.54E+05 | 7.86E+06 | 7.98E+05 |
| 10 | 1.71E+07 | 2.69E+07 | 3.56E+06 | 6.57E+05 | 1.59E+07 | 6.55E+06 |

**S8 Table. Participant’s average VLP abundances for each sample site in the oral cavity after sleep.**

| **Participant** | **Posterior buccal vestibule** | **Back of tongue** | **Gingivae** | **Palate** | **Molars** | **Tip of tongue** |
| --- | --- | --- | --- | --- | --- | --- |
| 1 | 2.07E+08 | 5.28E+08 | 1.49E+08 | 1.02E+08 | 3.44E+08 | 2.36E+07 |
| 2 | 5.44E+06 | 5.16E+07 | 1.82E+08 | 2.42E+06 | 1.80E+07 | 4.73E+06 |
| 3 | 9.47E+05 | 1.74E+07 | 5.38E+06 | 5.30E+05 | 1.28E+07 | 7.53E+05 |
| 4 | 1.05E+08 | 1.26E+08 | 5.39E+07 | 1.29E+07 | 2.34E+07 | 2.52E+07 |
| 5 | 3.01E+06 | 7.38E+06 | 9.25E+06 | 3.71E+06 | 2.92E+07 | 1.40E+06 |
| 6 | 1.20E+08 | 9.83E+07 | 9.39E+07 | 1.33E+07 | 1.00E+07 | 8.62E+07 |
| 7 | 3.11E+06 | 8.84E+06 | 5.71E+06 | 4.12E+05 | 1.52E+06 | 3.48E+05 |
| 8 | 6.38E+06 | 1.05E+07 | 1.04E+08 | 1.08E+06 | 4.39E+06 | 8.20E+06 |
| 9 | 1.07E+07 | 1.16E+07 | 3.19E+07 | 2.03E+06 | 6.07E+07 | 3.09E+06 |
| 10 | 1.08E+08 | 6.20E+07 | 2.87E+08 | 5.70E+06 | 6.16E+07 | 1.96E+07 |

| **Sample location** | **Bacterial percentage increase (± SEM)** | **VLP percentage increase (± SEM)** |
| --- | --- | --- |
| Posterior buccal vestibule | 2098%  (1464%) | 3638%  (3125%) |
| Back of tongue | 764%  (290%) | 416%  (240%) |
| Gingiva | 784%  (445%) | 1614%  (963%) |
| Palate | 1436%  (803%) | 700%  (323%) |
| Molars | 714%  (340%) | 662%  (180%) |
| Tip of tongue | 2391%  (1226%) | 2083%  (1911%) |

**S9 Table. Average percentage increase of bacteria and VLP within the paediatric oral cavity during sleep.** Error represents the standard error of the mean (SEM). Percentage increases were calculated by taking an average of each participant’s percentage increase/decrease for bacteria and VLPs for each sample location.
