# Supplementary figures and images for: The microbial abundance dynamics of the paediatric oral cavity before and after sleep
Source: J Oral Microbiol. 2020 Mar 30;12(1):1741254. doi: 10.1080/20002297.2020.1741254 (PMC7170375; doi:10.1080/20002297.2020.1741254)

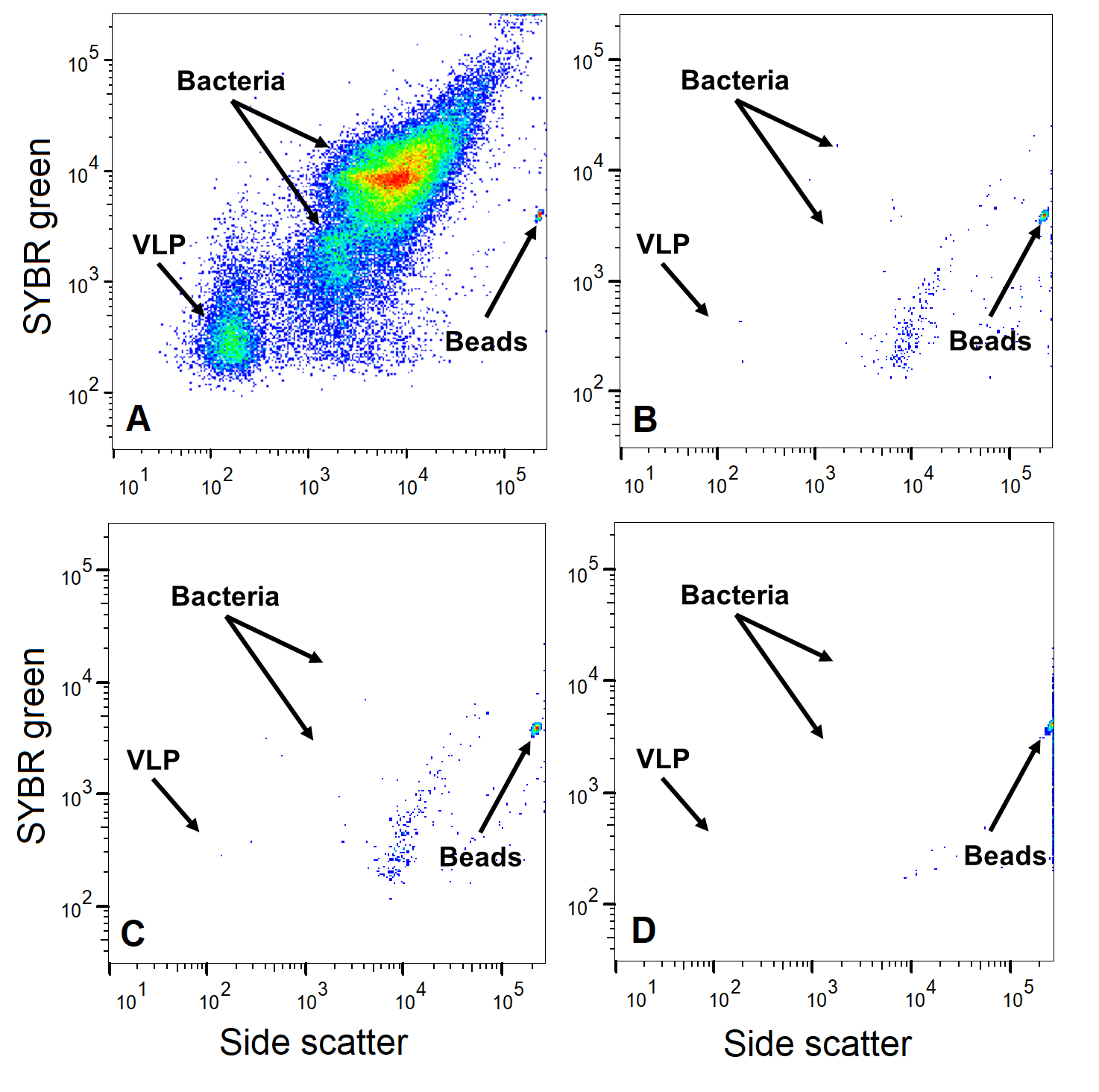

Supplement: Supplemental Material [file ZJOM_A_1741254_SM8966.zip › Supplementary/Figure_S1.tif]
